# Historical Changes in Histological Diagnosis of Lung Cancer

**DOI:** 10.2188/jea.JE20180037

**Published:** 2019-06-05

**Authors:** Mai Utada, Shuji Yonehara, Kotaro Ozasa

**Affiliations:** 1 Radiation Effects Research Foundation, Hiroshima, Japan; 2 JA Onomichi General Hospital, Hiroshima, Japan

**Keywords:** lung cancer, histological diagnosis, reproducibility

## Abstract

**Background:**

Histological classification of lung cancer is essential for investigations of carcinogenesis and treatment selection. We examined the temporal changes of lung cancer histological subtypes.

**Methods:**

Lung cancer cases diagnosed in the Life Span Study cohort between 1958 and 1999 were collected from tumor registries (TR), mainly consisting of population-based cancer registries. A total of 1,025 cases were histologically reviewed according to the World Health Organization 2004 Classification by a panel of pathologists (PP). Sensitivity and specificity of diagnoses in TR were calculated, assuming that the diagnosis by PP was the gold standard.

**Results:**

Sensitivity and specificity were 0.91 and 0.92 for adenocarcinoma (AD), respectively, and 0.92 and 0.94, respectively, for squamous cell carcinoma (SQ). They were similar for AD and SQ throughout the observation period. For small cell carcinoma (SM), sensitivity was low until about 1980 (0.47 in 1958–1969, and 0.61 in 1970–1979) and then became higher thereafter (0.98 in 1980–1989, and 0.95 in 1990–1999), whereas specificity was high during the whole period (range 0.99 to 1.00). Among 45 cases that were not reported as SM in TR but diagnosed as SM by PP, 16 cases were recorded as undifferentiated carcinoma in TR.

**Conclusion:**

Diagnosis of AD and SQ of lung cancer were generally consistent between TR records and PP review, but SMs tended to be coded as other histological types until the 1970s.

## INTRODUCTION

Lung cancer has been the most common cancer worldwide for several decades.^1^ In Japan, lung cancer mortality increased after World War II and is currently the leading cause of death from cancer. Rates have decreased in males since the late 1990s but have remained relatively stable in females.^2^ Histological classification is essential for pathological investigation of carcinogenesis and has recently become critical for the selection of treatment methods based on different sensitivities to chemotherapy and radiotherapy by subtype of lung cancer.^3^ Several studies indicated high inter-observer agreement of histological diagnoses and high reproducibility of histological types reported to cancer registries in independent review.^4^^–^^6^ We believe that it is important from the viewpoint of descriptive epidemiology to show a long-term trend in histological subtypes of lung cancer based on the current diagnostic criteria. The results are thought to be helpful to explore emergence of novel subtypes, historical impact of risk factors on specific subtypes (eg, not only for tobacco consumption and lung cancer as a whole, but also for the associations between tobacco product types and specific histological subtypes), and other investigations on historical aspects.

The aim of this study was to explore the temporal change in agreement between histological diagnosis of lung cancer reported to tumor registries and that determined by a panel of pathologists.

## MATERIALS AND METHODS

Population-based cancer registries were initiated in Hiroshima in 1957 and in Nagasaki in 1958. A system for direct reporting of histological diagnosis from pathologists in local hospitals was initiated in Hiroshima in 1973 and in Nagasaki in 1974 and were called “tissue registries”. In the population-based registries and the tissue registries, histological diagnoses were coded using the International Classification of Diseases for Oncology (ICD-O).^7^^–^^9^ For simplification, we call these two sources tumor registries (TRs). We have collected information on incident lung cancer cases and their coded histological diagnoses occurring among members of the Life Span Study (LSS) cohort between 1958 and 1999, together with some supplementary sources of information available to the Radiation Effects Research Foundation (RERF), an institution dedicated to studying the long-term health effects among the survivors of the atomic bombings in Hiroshima and Nagasaki, Japan. A comprehensive histological review of those lung cancer cases was conducted for radiation risk analyses in the late 1990s to early 2000s.^10^^,^^11^ Microscopic glass slides and/or paraffin blocks from these cases were borrowed from local hospitals and those materials were reviewed and diagnosed according to the World Health Organization (WHO) 2004 classification^12^ by a panel of three pathologists (PP). Members of the PP reviewed hematoxylin and eosin (HE)-stained slides independently and, if necessary, specially stained materials made from paraffin blocks were reviewed. After discussion, a unified diagnosis was reached.

Among available lung cancer cases in the study,^10^^,^^11^ 1,025 cases were diagnosed with histological information by both the TR and the PP. Histological types were aggregated into adenocarcinoma (AD), squamous cell carcinoma (SQ), small cell carcinoma (SM), and others. We calculated sensitivity and specificity of TR diagnoses, assuming that the diagnosis by PP was the gold standard. Ninety five percent confidence intervals were estimated based on the normal distribution.

This study was based on data collected from RERF Research Protocols 1-75, 18-61, and 1-94, which follow the LSS cohort, collect cancer incidence information from local cancer and tissue registries, and investigate radiation risks of histologically diagnosed lung cancers in the LSS. All protocols were approved by RERF’s institutional review board and the relevant cancer and tissue registry offices in Hiroshima and Nagasaki.

## RESULTS

Comparisons between ICD-O codes recorded in the TR and by the PP are shown in Table 1. For all cases, the sensitivity and specificity of AD diagnoses was 0.91 (431/475) and 0.92 (504/550), respectively (Table 2). The respective figures were 0.92 (244/265) and 0.94 (712/760) for SQ and 0.73 (120/165) and 0.99 (854/860) for SM.

**Table 1.** Comparison between diagnoses recorded in tumor registries and those reviewed by panel of pathologists

Records in tumor registries	Diagnosis by panel of pathologists (WHO 2004)				

	AD	SQ	SM	Others	Total
Adenocarcinoma (AD)	431	7	6	33	477
Squamous cell carcinoma (SQ)	12	244	11	25	292
Small cell carcinoma (SM)	1	4	120	1	126
Others	31	10	28	61	130
Total	475	265	165	120	1,025

WHO, World Health Organization.

**Table 2.** Sensitivity and specificity of lung cancer by histological type in tumor registry contrasted with that by a panel of pathologists

	Year of diagnosis	Sensitivity	95% CI		Specificity	95% CI	
	
			Lower	Upper		Lower	Upper
AD	1958–1969	0.88	0.82	0.95	0.93	0.89	0.97
	1970–1979	0.85	0.78	0.93	0.92	0.88	0.97
	1980–1989	0.92	0.88	0.97	0.91	0.86	0.96
	1990–1999	0.94	0.90	0.97	0.90	0.84	0.95
	Total	0.91	0.88	0.93	0.92	0.89	0.94

SQ	1958–1969	0.93	0.87	0.99	0.88	0.83	0.93
	1970–1979	0.95	0.90	1.00	0.92	0.88	0.96
	1980–1989	0.87	0.79	0.95	0.95	0.93	0.98
	1990–1999	0.93	0.87	1.00	0.97	0.95	0.99
	Total	0.92	0.89	0.95	0.94	0.92	0.95

SM	1958–1969	0.47	0.34	0.60	0.99	0.98	1.01
	1970–1979	0.61	0.44	0.77	1.00	1.00	1.00
	1980–1989	0.98	0.93	1.02	0.99	0.97	1.00
	1990–1999	0.95	0.87	1.02	0.99	0.98	1.00
	Total	0.73	0.66	0.80	0.99	0.99	1.00

AD, adenocarcinoma; CI, confidence interval; SM, small cell carcinoma; SQ, squamous cell carcinoma.

Detailed comparison between TR and PP are shown in eTable 1. In total, 10 cases were not classified with any specific diagnosis by the PP. Forty-four cases that were not recorded as AD in the TR, but diagnosed as AD by the PP (hereinafter, AD cases with TR− and PP+, and in a similar way for other combinations), included 13 cases of large cell carcinoma not otherwise specified (NOS), and 12 cases of squamous cell carcinoma NOS in the TR. In contrast, 46 AD cases with TR+ and PP− included 10 cases of adenosquamous carcinoma, and 9 cases of large cell carcinoma diagnosed by the PP. In a similar fashion, 21 SQ cases with TR− and PP+ included 7 cases of adenocarcinoma NOS in the TR, while 48 SQ cases with TR+ and PP− included 12 cases of adenocarcinoma and 9 cases of large cell carcinoma diagnosed by the PP. Forty-five SM cases with TR− and PP+ included 16 cases of undifferentiated carcinoma NOS, 11 cases of squamous cell carcinoma NOS, 6 cases of adenocarcinoma, and 5 cases of anaplastic carcinoma in the TR, while 6 SM cases with TR+ and PP− included 4 cases of squamous cell carcinoma diagnosed by the PP.

Sensitivity and specificity for AD were similar by period of diagnosis (range 0.85 to 0.94 for sensitivity and 0.90 to 0.93 for specificity; Table 2) and for SQ (range 0.87 to 0.95 and 0.88 to 0.97, respectively). For SM, the sensitivity was low until about 1980 (0.47 in 1958–1969 and 0.61 in 1970–1979) and then was high thereafter (0.98 in 1980–1989 and 0.95 in 1990–1999). On the other hand, specificity for SM was high during the whole period (range 0.99 to 1.00).

## DISCUSSION

We evaluated the temporal changes in consistency of histological diagnoses of lung cancer between TR records and PP review. Diagnoses of AD and SQ in the TR was generally consistent with the PP throughout the observation period from 1958 to 1999. SM tended to be reported in the TR as other histological types until the 1970s, often as “undifferentiated carcinoma”. On the other hand, cancers of other histological types were less likely to be reported as SM throughout the study period. After the 1980s, diagnoses of the three major histological types between TR records and PP review were very consistent. This finding was similar to a previous study using data from the Iowa Cancer Registry.^6^

One plausible reason of improved sensitivity of SM after the 1980s was the publication of the first edition of “Classification of Lung Carcinoma” by the Japan Lung Cancer Society in 1978,^13^ which included diagnostic criteria for SM that were similar to the WHO classification criteria. Before that time, small cell carcinoma and large cell carcinoma were often included in the category of undifferentiated or anaplastic carcinoma. In early practice, the terms “undifferentiated small cell carcinoma” and “small cell anaplastic carcinoma” were historically used.^3^ Of 21 cases that were recorded as undifferentiated carcinoma and anaplastic carcinoma in the TR but diagnosed as SM by PP review (eTable 1), 20 were reported prior to the 1980s. Those diagnoses may not have disagreed with the current concept of SM. Introduction of special staining procedure in practice is thought to improve the consistency of diagnosis between TR records and PP review.

Some cases classified as SQ and AD in the TR records but as SM by the PP may have been “combined small cell carcinoma,” which has components of both SM and non-SM and would be classified as SM using the recent diagnostic criteria.^3^^,^^12^ There were two possible reasons for the inconsistent diagnoses in the past. First, SQ or AD components were previously prioritized regardless of the SM component. Second, the SM component may have been missed or not noted from the slide at the time of the diagnosis. The former was plausible because no SM cases were recorded by the TR after the 1980s as SQ or AD by the PP.

Specially stained specimens from paraffin blocks, in addition to HE-stained slides, were sometimes used in the PP review to identify the histological type for suspected cases. This process was thought to have enabled more accurate diagnoses, especially for large cell carcinoma, of which the diagnosis needs to exclude AD, SQ, or SM features. The changes in definition of histological types were also thought to have altered the diagnosis of AD, SQ, and adenosquamous carcinoma. Adenosquamous carcinoma was defined as having both AD and SQ components with each component comprising at least 10% of the tumor for the first time in the WHO 2004 classification.^12^ Before that, some cases compatible with adenosquamous carcinoma in the WHO 2004 classification might have been simply diagnosed as AD or SQ.

The strength of this study was that all lung cancer cases with available histological specimens were diagnosed using the WHO 2004 classification. Each diagnosis reached by the panel of three pathologists was considered the gold standard. The major reason that 10 cases could not be classified as any specific diagnosis was the small size of specimens obtained by transbronchial lung biopsy or strong autolysis of specimens.

Prior to the 1980s, histological classifications were primarily derived from morphologic features because the etiology and pathogenesis of cancers were not sufficiently understood to drive classification. Modern techniques allow histological classifications to be based on stages of cell differentiation, characteristics of chromosomes and genes, and surface markers that are thought to associate with carcinogenic pathways and responsiveness to therapies. Consequently, the classification system was updated in 2015, which was after completion of the data gathered for these studies.^10^^,^^11^ In the new classification system, SM is explicitly defined as a subtype of neuroendocrine tumors.^3^^,^^14^ Large cell neuroendocrine carcinoma (LCNEC) was first introduced as a subtype of large cell carcinoma in the WHO 2004 classification but is included in a subgroup of neuroendocrine tumors as well as SM in the WHO 2015 classification.^3^^,^^14^ Nonetheless, differential diagnosis of LCNEC from major histological types of AD and SQ is thought to be more important. Therefore, we do not believe that the recent update would markedly influence the results of this study, but further studies for detailed differential diagnoses may be required.

In conclusion, diagnoses of AD and SQ of lung cancer were thought to be consistent between TR records reported in the past and a PP review using the WHO 2004 classification system throughout the study period. Changes in the diagnostic classification system were not thought to influence these results. In contrast, SM tended to be recorded as other histological types prior to the 1980s, as the concept and criteria for SM was not distributed among local pathologists until after the 1978 publication of a Japanese classification system. The consistency of histological diagnoses between records in TR and the PP, particularly in recent years, was thought to be due to progress in understanding the underlying etiology and pathogenesis of lung cancer.

## APPENDIX A. SUPPLEMENTARY DATA

eTable 1. Comparison between diagnoses recorded in tumor registries and those reviewed by by panel of pathologists (detailed classification)

## References

[1] GLOBOCAN 2012. Estimated cancer incidence, mortality and prevalence worldwide in 2012. http://globocan.iarc.fr/Pages/fact_sheets_cancer.aspx; Accessed 12.01.2017.

[2] Katanoda K, Hori M, Matsuda T, An updated report on the trends in cancer incidence and mortality in Japan, 1958–2013. Jpn J Clin Oncol. 2015;45:390–401. 10.1093/jjco/hyv002 25637502

[3] World Health Organization. *WHO Classification of Tumours of the Lung, Pleura, Thymus and Heart*. Lyon: IARC Press; 2015. 10.1097/JTO.0000000000000663 26291007

[4] Paech DC, Weston AR, Pavlakis N, A systematic review of the interobserver variability for histology in the differentiation between squamous and nonsquamous non-small cell lung cancer. J Thorac Oncol. 2011;6:55–63. 10.1097/JTO.0b013e3181fc0878 21107286

[5] Yamamoto S, Sobue T, Yamaguchi N, Reproducibility of diagnosis and its influence on the distribution of lung cancer by histologic type in Osaka, Japan. Jpn J Cancer Res. 2000;91:1–8. 10.1111/j.1349-7006.2000.tb00853.x 10744038 PMC5926225

[6] Field RW, Smith BJ, Platz CE, Lung cancer histologic type in the surveillance, epidemiology, and end results registry versus independent review. J Natl Cancer Inst. 2004;96:1105–1107. 10.1093/jnci/djh189 15265973

[7] World Health Organization. *International Classification of Diseases for Oncology, 3rd Edition (ICD-O-3)*. Geneva: World Health Organization; 2000.

[8] World Health Organization. *International Classification of Diseases for Oncology, 2nd Edition (ICD-O-2)*. Geneva: World Health Organization; 1990.

[9] World Health Organization. *International Classification of Diseases for Oncology*. Geneva: World Health Organization; 1975.

[10] Furukawa K, Preston DL, Lönn S, Radiation and smoking effects on lung cancer incidence among atomic bomb survivors. Radiat Res. 2010;174:72–82. 10.1667/RR2083.1 20681801 PMC3857029

[11] Egawa H, Furukawa K, Preston D, Radiation and smoking effects on lung cancer incidence by histological types among atomic bomb survivors. Radiat Res. 2012;178:191–201. 10.1667/RR2819.1 22862780 PMC4031371

[12] World Health Organization. *Pathology and genetics of tumours of the lung, pleura, thymus and heart*. Lyon: IARC Press; 2004.

[13] Japan Lung Cancer Society. *Classification of Lung Carcinoma*. 8th ed. Tokyo: Kanehara shuppan; 2017.

[14] Travis WD, Brambilla E, Nicholson AG, The 2015 World Health Organization Classification of Lung Tumors: Impact of Genetic, Clinical and Radiologic Advances Since the 2004 Classification. J Thorac Oncol. 2015;10:1243–1260. 10.1097/JTO.0000000000000630 26291008

