# Supplementary material for: Historical Changes in Histological Diagnosis of Lung Cancer
Source: J Epidemiol. 2019 Jun 5;29(6):238–40. doi: 10.2188/jea.JE20180037 (PMC6522388; doi:10.2188/jea.JE20180037)
Supplement: Supplementary file 1 [file je-29-238-s001.pdf]

eTable 1. Comparison between diagnoses recorded in tumor registries and those reviewed by by panel of pathologists (detailed classification)

			Diagnosis by pathologist panel (WHO 2004)																
			AD						SQ	SM		Others							
			Adenocarcinoma (NOS)	Adenocarcinoma, mixed subtype	Acinar adenocarcinoma	Papillary adenocarcinoma	Bronchiololalveolar carcinoma	Solid adenocarcinoma with mucin production	Squamous cell carcinoma	Small cell carcinoma	Combined small cell carcinoma	Large cell carcinoma	Adenosquamous carcinoma	Sarcomatoid carcinoma	Carcinoid tumors	Salivary gland tumors	Not otherwise specified	Total
			8140	8255	8550	8260	8250, 8250-8254	8230, 8333, 8480, 8470, 8490	8070, 8052, 8084, 8073, 8083	8041	8045	8012, 8013, 8123, 8082, 8310, 8014	8560	8033, 8022, 8032, 8031, 8980, 8972	8240, 8249	8430, 8200, 8562	-	
ICD-O morphology codes in tumor registries	AD	8140	Adenocarcinoma, NOS	78	153	30	8	2	3	7	3	2	7	8	6	2	2	3	314
		8211	Tubular adenocarcinoma, NOS	0	15	9	0	0	2	0	1	0	2	2	0	0	0	0	31
		8230	Solid carcinoma, NOS	0	0	1	0	0	0	0	0	0	0	0	0	0	0	0	1
		8250	Bronchiolo-alveolar adenocarcinoma, NOS	2	18	0	6	1	1	0	0	0	0	0	0	0	0	0	28
		8251	Alveolar adenocarcinoma	1	0	0	2	0	0	0	0	0	0	0	0	0	0	0	3
		8260	Papillary adenocarcinoma, NOS	6	64	0	22	0	1	0	0	0	0	0	1	0	0	0	94
		8480	Mucinous adenocarcinoma	0	2	1	0	0	0	0	0	0	0	0	0	0	0	0	3
		8490	Signet ring cell carcinoma	1	0	1	0	0	0	0	0	0	0	0	0	0	0	0	2
		8510	Medullary carcinoma, NOS	0	0	1	0	0	0	0	0	0	0	0	0	0	0	0	1
	SQ	8070	Squamous cell carcinoma, NOS	3	5	3	0	0	1	240	5	6	9	5	8	0	0	3	288
		8071	Squamous cell carcinoma, keratinizing, NOS	0	0	0	0	0	0	3	0	0	0	0	0	0	0	0	3
		8072	Squamous cell carcinoma, large cell, nonkeratinizing, NOS	0	0	0	0	0	0	1	0	0	0	0	0	0	0	0	1
	SM	8041	Small cell carcinoma, NOS	1	0	0	0	0	0	4	74	9	0	0	1	0	0	0	89
		8042	Oat cell carcinoma	0	0	0	0	0	0	0	29	3	0	0	0	0	0	0	32
		8044	Small cell carcinoma, intermediate cell	0	0	0	0	0	0	0	5	0	0	0	0	0	0	0	5
	Ohters	8000	Neoplasm, malignant	0	0	0	0	0	0	0	1	0	0	0	0	0	0	0	1
		8010	Carcinoma, NOS	2	1	0	0	0	0	2	4	0	2	0	0	0	0	2	13
		8012	Large cell carcinoma, NOS	2	3	6	0	0	2	2	1	0	5	0	8	0	0	1	30
		8020	Carcinoma, undifferentiated, NOS	2	0	0	0	0	0	2	14	2	6	0	4	0	0	1	31
		8021	Carcinoma, anaplastic, NOS	0	2	1	0	0	0	2	3	2	4	0	1	0	0	0	15
		8022	Pleomorphic carcinoma	0	0	0	0	0	0	0	0	0	1	0	1	0	0	0	2
		8031	Giant cell carcinoma	1	0	0	0	0	0	0	0	0	0	0	2	0	0	0	3
		8046	Non-small cell carcinoma	1	0	0	0	0	0	0	0	0	0	0	0	0	0	0	1
		8231	Carcinoma simplex	0	0	0	0	0	0	0	0	0	0	0	0	1	0	0	1
		8240	Carcinoid tumor, NOS	0	0	0	0	0	0	0	0	0	0	0	0	6	0	0	6
		8246	Neuroendocrine carcinoma, NOS	0	0	0	0	0	0	0	1	0	0	0	0	0	0	0	1
		8430	Mucoepidermoid carcinoma	0	0	1	0	0	0	0	0	0	0	0	0	0	0	0	1
		8560	Adenosquamous carcinoma	0	5	2	0	0	0	2	0	0	0	15	0	0	0	0	24
		8570	Adenocarcinoma with squamous metaplasia	0	0	0	0	0	0	0	0	0	0	1	0	0	0	0	1
	Total			100	268	56	38	3	10	265	141	24	36	31	32	9	2	10	1025

AD, adenocarcinoma; NOS, not otherwise specified; SM, small cell carcinoma; SQ, squamous cell carcinoma; WHO, World Health Organization.
